# Mapping Arctic cetaceans from space: A case study for beluga and narwhal

**DOI:** 10.1371/journal.pone.0254380

**Published:** 2021-08-04

**Authors:** Bertrand Charry, Emily Tissier, John Iacozza, Marianne Marcoux, Cortney A. Watt

**Affiliations:** 1 Whale Seeker, Montreal, Quebec, Canada; 2 Centre for Earth Observation Science, Department of Environment and Geography, University of Manitoba, Winnipeg, Manitoba, Canada; 3 Arctic Aquatic Research Division, Fisheries and Oceans Canada, Winnipeg, Manitoba, Canada; 4 Department of Biological Sciences, University of Manitoba, Winnipeg, Manitoba, Canada; Wildlife Conservation Society Canada, CANADA

## Abstract

Emergence of new technologies in remote sensing give scientists a new way to detect and monitor wildlife populations. In this study we assess the ability to detect and classify two emblematic Arctic cetaceans, the narwhal (*Monodon monoceros*) and beluga whale (*Delphinapterus leucas*), using very high-resolution (VHR) satellite imagery. We analyzed 12 VHR images acquired in August 2017 and 2019, collected by the WorldView-3 satellite, which has a maximum resolution of 0.31 m per pixel. The images covered Clearwater Fiord (138.8 km^2^), an area on eastern Baffin Island, Canada where belugas spend a large part of the summer, and Tremblay Sound (127.0 km^2^), a narrow water body located on the north shore of Baffin Island that is used by narwhals during the open water season. A total of 292 beluga whales and 109 narwhals were detected in the images. This study contributes to our understanding of Arctic cetacean distribution and highlights the capabilities of using satellite imagery to detect marine mammals.

## Introduction

Emergence of new technologies in remote sensing are pushing forward the fields of ecology and conservation by expanding the range and scale at which researchers can conduct their studies and monitor populations [1–4]. Remote sensing imagery, whether obtained from satellites, airplanes, or remotely piloted aircraft systems (RPAS), can capture demographic information, spatial distribution and habitat selection of animal populations with minimum observer bias [4–7]. Remote sensing imagery can also be collected at any time of the year; and offers a less invasive method for monitoring large mammals [2]. However, some important hurdles in advancing population monitoring through remote imagery include: our ability to detect and correctly classify animals from different spatial resolutions, collecting new imagery in remote locations or extreme conditions, and obtaining imagery of animals that move and are distributed across large areas at a resolution that is able to detect animals [4, 8, 9].

To face this last challenge, researchers have been relying on aerial sampling methods, such as aircrafts or RPAS, employing predetermined survey transects to limit the probability of counting individuals multiple times when monitoring wildlife [10]. However, this aerial monitoring method is not ideal for many cetacean species characterized by long distance seasonal migrations and highly aggregated distributions across large spatial scales [11]. Their capacity to move long distances in a short amount of time adds another level of difficultly for planning full-coverage population surveys when using traditional aerial methods [12].

In recent years, imagery from very high-resolution (VHR) satellites have successfully been used as a non-invasive method to monitor remote wildlife species both on land and water such as the Central Arctic Caribou Herd (*Rangifer tarandus granti*) migration on the north slope of Alaska (Satellite Imaging Corporation, 2018), southern right whales (*Eubalaena australis*), hauled-out Weddell seals (*Leptonychotes weddellii*), and polar bears (*Ursus marinus*) [8, 13, 14]. The low Earth orbit of VHR satellites and their nearly polar orbit permit the acquisition of sub-meter resolution imagery and high polar coverage. In addition, the sun-synchronous orbit of VHR satellites maintains the highest constant illumination level for a given season, facilitating image interpretation and comparison over time [15, 16].

Monitoring Arctic cetaceans is particularly difficult due to the remoteness and vastness of the Arctic environment. The Arctic Ocean spreads over 14 million square kilometers and is home to many cetacean species including the bowhead whale (*Balaena mysticetus*), beluga (*Delphinapterus leucas*), and narwhal (*Monodon monoceros*) which are year-round residents [17]. The limited light period, open water season, and cryptic behavior of cetaceans spending most of their time underwater, greatly diminish our opportunities to observe and monitor them. Thus, the opportunity to use satellite imagery as a new, noninvasive method for monitoring Arctic cetaceans is timely [18] and highly advantageous for conservationists, managers, and researchers.

To date, researchers have been able to identify large whales (>10 m) such as right whales, fin whales (*Balaenoptera physalus*), humpback whales (*Megaptera novaeangliae*) and gray whales (*Eschrichtius robustus*) from VHR satellite imagery [2, 14]. However, to our knowledge, it has not been tested on smaller cetacean species (< 10 m). Our study is the first to detect medium-sized cetaceans from satellite imagery by investigating two populations of Arctic whales, the Cumberland Sound beluga and Baffin Bay narwhal populations, to determine whether aggregations and/or individuals could be detected using satellite imagery. Beluga whales and narwhals are highly social animals that aggregate in large numbers across time and space [19, 20]. Both species are found in Arctic and sub-Arctic waters, and are characterized as medium sized whales reaching an average size of 3 to 5 m in length during adulthood, with a rounded forehead, and absence of a dorsal fin which is replaced by a ridge [21, 22]. These species do, however, differ in coloration patterns. Beluga whales are born with a grey-cream color but change to a dark brown or slate grey shortly after birth; then whales lighten from gray to white as they age with most whales becoming completely white in their mid-teens [21]. Narwhals, on the other hand, are uniform grey as young and then develop a mottled white and black coloration as they age [22]. With age, the mottling becomes dominated by white pigmentation [22]. Moreover, narwhals show a sexual dimorphism with males being larger than females and displaying a tusk, that can reach nearly 3.0 m in length, that erupts when males are roughly one year old (when they measure between 2.0 and 2.5 m), whereas females generally lack a tusk [23–26].

The two species, narwhal and beluga, present interesting characteristics that make them ideal subjects to assess the feasibility of detecting them from space. Both species are highly gregarious with known summer distributions [27, 28] and very little overlap with other cetacean species, which makes species identification more reliable. However, narwhals, with their mottled grey pigmentation [22], present a more limited color contrast with their surrounding environment compared to the pure white coloration of beluga whales [21], which offers a stark contrast with their environment making them an easier target to detect from space. The objective of this study was to evaluate the feasibility of detecting narwhal and beluga from satellite imagery which could assist with monitoring their abundance and distribution in the future.

## Materials and methods

### Beluga population and study area

**Fig 1:**
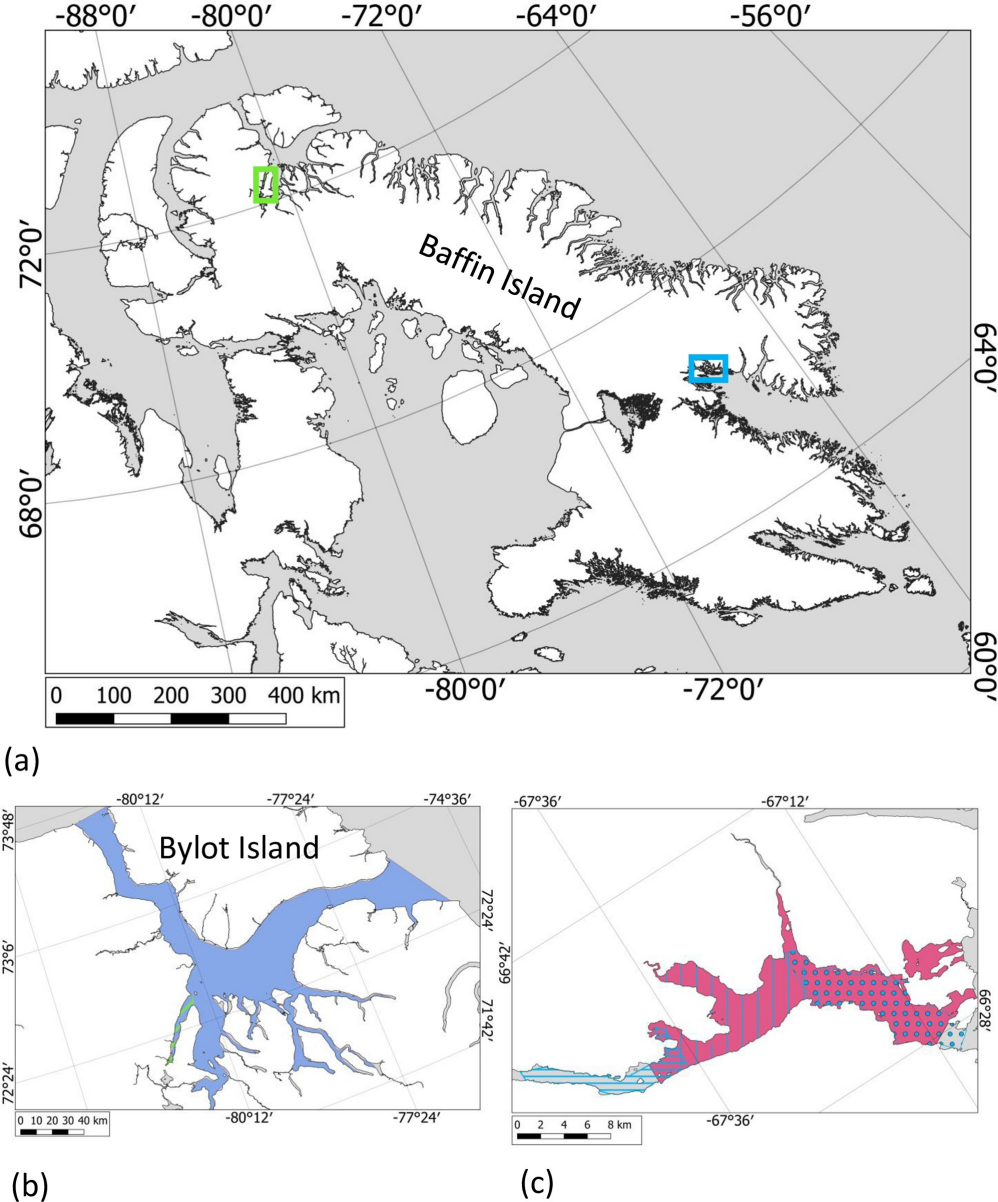
Location of study area for a) narwhal (in green) and beluga (in blue). Zoom in of b) Tremblay Sound with image footprint in green taken on August 02, 2017 and distribution of narwhal in blue from aerial survey in 2016 [37]) c) Clearwater Fiord with blue vertical lines, dots and horizontal lines representing images footprint taken on August 4, August 6 and August 09, 2019 respectively, on Baffin Island, Canada and beluga August distributions indicated in purple (distribution for 14 beluga tagged in August [30].

The Cumberland Sound beluga population is approximately 1,100 individuals [29] (Fig 1). Telemetry information from 14 individuals from this population suggests they spend their entire lifecycle in Cumberland Sound, an Arctic waterway located in the southeastern part of Baffin Island between Hall peninsula and Cumberland peninsula in Nunavut, Canada. The sound is a large bay composed of multiple fiords [30]. During summer months, during the open water season a large portion of the population aggregates and stays in Clearwater Fiord, located at the northern end of the sound (66°34’2.8” N, 67°26’16.8” W; Fig 1) [29, 31]. Narwhals are not typically present in Clearwater Fiord in August.

### Narwhal population and study area

The Baffin Bay narwhal population is the largest narwhal population in the world (> 140,000 narwhals; [32]), whose range spans Canadian and Greenlandic waters. They are known to conduct long-distance migrations in large groups, spending winters in dense pack-ice and summers in fiords and bays of the Canadian Arctic Archipelago [27, 33–36]. Narwhals are believed to seek areas of complex shoreline in part to avoid predation from killer whales [36]. During the summer, they can aggregate in large herds of hundreds of individuals [19]. For this study, we focused our effort on the Eclipse Sound summer aggregation, that is visited by an estimated 12,000 narwhals during the ice free season (Fig 1A [37]).

Tremblay Sound (72°25’5” N, 80°59’55.9” W; Fig 1B), a narrow water body, is located on the north shore of Baffin Island, Nunavut, Canada within the distribution of the Eclipse Sound summer aggregation. Narwhals mostly stay within the Eclipse Sound area but can move in and out of Tremblay Sound on a daily basis [35]. As a result, aerial surveys designed to estimate the abundance of the narwhal summer aggregation aim to cover the entire area within a few days [37]. Beluga are rarely seen in this area [19, 33].

### Satellite imagery selection and acquisition

The WorldView-3 (WV3) satellite is a VHR commercial Earth observing system owned by DigitalGlobe. Launched in 2014, WV3 images the Earth at an altitude of 670 km in a sun-synchronous orbit, passing over the equator at 13:30 (local time) in the descending path. The swath width of the imagery is 13.1 km at nadir. A single band panchromatic image from WV3 has a spatial resolution of 0.31 m at nadir; while multispectral imagery collected using eight different spectral bands from visible to near infrared has a resolution of 1.24 m.

The satellite images, collected from the WV3 satellite, covered a total area of 138.8 km^2^ of Clearwater Fiord in August 2019. The date of satellite image acquisition was based on likelihood to observe large numbers of belugas in the study area with calm sea-state (maximum Beaufort Sea State 3) and <15% cloud cover (Table 1). Four images were collected middays on 4 August, three images on 6 August, and two images on 9 August, 2019 (Table 1). These images were taken using WV3 sensors at an approximately one second acquisition time interval. Images were stitched together by L3Harris Geospatial from Digital Globe to remove any spatial overlap.

**Table 1:** WorldView-3 satellite images (0.31 m resolution) taken in Nunavut, Canada in 2017 and 2019.

Location	Species	Date	# of images	Cloud cover (%)	Beaufort Sea State	Whales detected	Uncertain detections	Targets undetected by observer A	Targets undetected by observer B	Whale agreement
Clearwater Fiord	Beluga	2019-08-04	4	14.0	1–2	276	68	48	19	248
Clearwater Fiord	Beluga	2019-08-06	3	9.6	1–3	10	18	7	11	8
Clearwater Fiord	Beluga	2019-08-09	2	2.6	1–3	6	0	0	0	6
Tremblay Sound	Narwhal	2017-08-02	3	0.0	1	109	117	31	33	99

*all images were acquired from L3 Harris Geospatial.

Three satellite images of Tremblay Sound, acquired from WV3 at ~22 and 13 second intervals on 2 August, 2017, covered the entire study area (127 km^2^). The date was carefully chosen based on the absence of sea ice, a calm sea state (Beaufort Sea State 1; Table 1), and observations of narwhals by the Department of Fisheries and Oceans Canada (DFO) in Tremblay Sound on 1 and 3 August, 2017.

All satellite imagery for this study was acquired through L3Harris Geospatial from Digital Globe with initial processing including ortho-rectification, color balance, and data preparation to GeoTIFF format and an Enhanced Compression Wavelet (ECW) file.

### Satellite image analysis

Two observers, A and B, gained experience analyzing satellite images for beluga from 18 WV3 images of Clearwater Fiord in 2017, prior to this study.

The panchromatic images from WV3 were interpreted using QGIS 3.10. Using a 2.5 km^2^ grid, the images were read independently by the two observers from left to right at a scale of 1:535. Each observer recorded beluga whales or narwhals as georeferenced point stored data layers. When an observation was considered uncertain by an observer this observation was recorded in a new georeferenced point layer named “uncertain”. In this study the term agreement signifies that both observers identified the same object of interest, at the same location, and gave the same classification of either a whale or uncertain. Disagreement is used when an object was only seen by one observer, or when the two observers disagreed on the classification of the object as a whale or uncertain.

To determine if results obtained by observer A and B could be repeated by other marine mammal observers with no prior experience in whale detection from satellite imagery a third observer, who has had substantial experience reading aerial photos of belugas in the same area but was new to satellite imagery analysis, examined 23 cropped satellite image sections of 6.5 km^2^ taken in Clearwater Fiord. The cropped images were selected to provide a range of number and quality of detections in the sections.

After the full visual analysis of beluga whale imagery was completed, a subsection (1:177 scale) of what both observers agreed were surface and submerged belugas were selected for pansharpening to determine whether this method could enhance animal detection. Three different pansharpening algorithms were visually assessed (Fig 3) on surface and submerged belugas (submerged animals were identified by observer A and B based on darker color shades and no visual of the full body outline of individual whales): Fast Intensity-Hue-Saturation (FIHS), Brovey Transform (BT), and Additive Wavelet Transform (AWT). Pansharpening is an image enhancement technique that merges the high spatial resolution panchromatic information with the lower resolution multispectral bands; creating a higher resolution multispectral image. In all three pansharpening methods used in this study, the RGB components of the color image are transformed to adjust intensity (i.e. brightness), hue (i.e. dominant wavelength) and saturation (i.e. purity of color). In a FIHS algorithm, intensity component is replaced by the panchromatic image that is stretched so the mean and variance matches the intensity component. This method allows for quick processing of large data volumes. In the BT algorithm, each of the RGB bands are multiplied by the product of the panchromatic image divided by the sum of the RGB bands [38]. In the AWT algorithm, a gaussian low pass filter is applied to the panchromatic image, creating a lower resolution panchromatic image. Both the low and high resolution panchromatic images are then transformed into n wavelet planes and the intensity component of the original image is added to the difference between the wavelet planes [39]. In all three algorithms, the transformed image is then re-projected back into RGB color space. Lastly, the observers visually assessed all combinations of 3-color bands to determine which combination provides the best contrast between belugas and the water.

We were unable to test pan sharpening algorithms on narwhals due to corruption of the multispectral data for these images.

## Results

**Fig 2:**
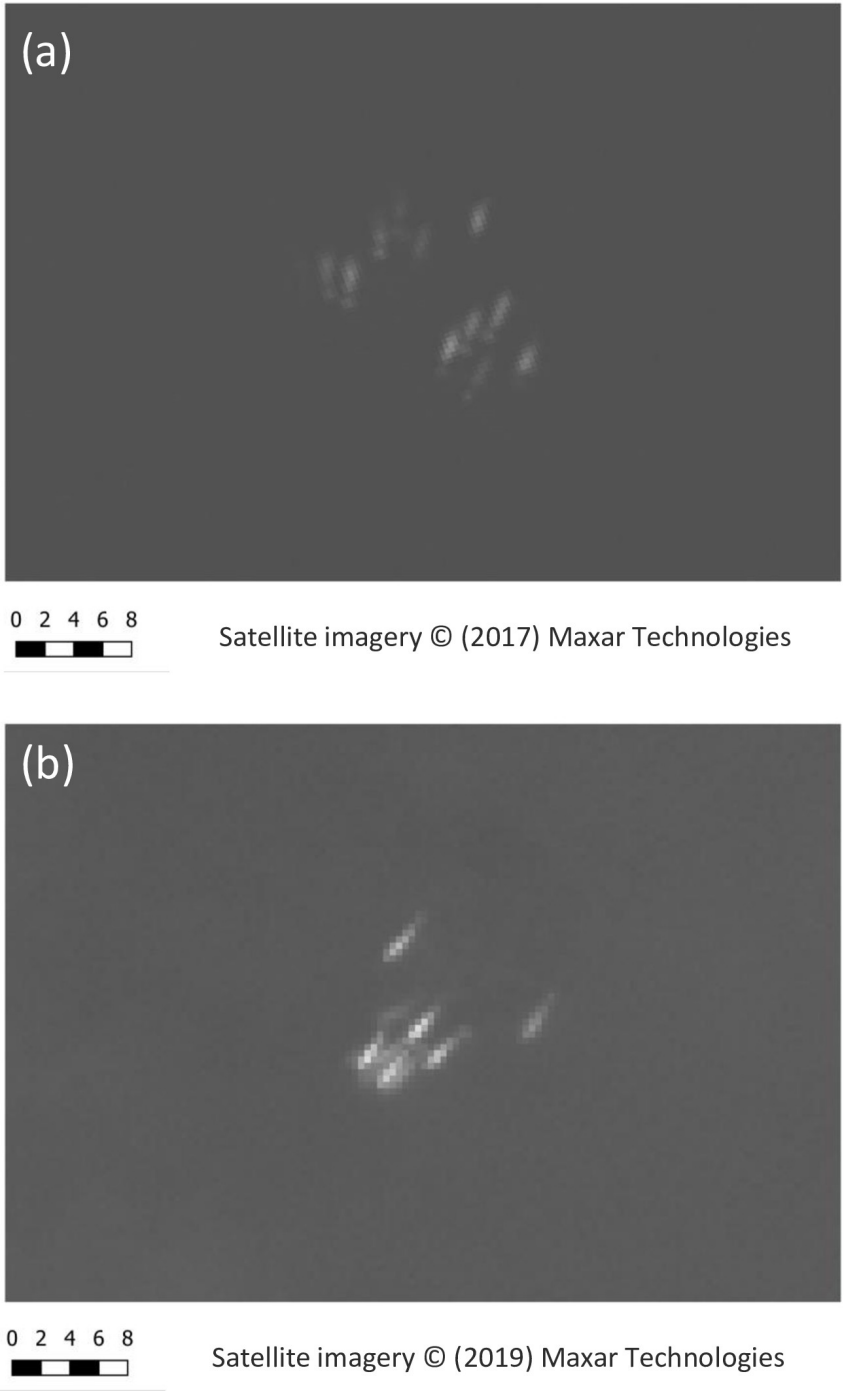
Narwhal (a) and beluga (b) individuals detected from panchromatic WorldView-3 satellite images at a 1:265 scale. Republished under a CC BY license, with permission from Maxar Technologies, original copyright 2017 and 2019.

Beluga and narwhal individuals were clearly detectable from satellite imagery taken from the WV3 satellite (Fig 2).

From the satellite images acquired of Clearwater Fiord in 2019, 378 targets were detected combined between the two observers. Most targets were observed on 4 August with 276 belugas and 68 uncertain targets detected. Only 10 belugas and 18 uncertain were observed on 6 August, and six belugas on 9 August. Of the total targets recorded in all three days, 7.9% (n = 30) were undetected by observer B, and 14.5% (n = 55) were undetected by observer A. Of the detected targets by both observers the mean percentage classification disagreement was 7% (n = 27) where an observer classified a target as a whale and the other observer classified the target as uncertain. The whale agreement between both observers was 89.7% (n = 262).

Total targets from narwhal images taken on 2 August, 2017 were determined using the same criteria as for the beluga imagery described above, yielding 226 total targets (including uncertain observations). Of this total, 14.6% (n = 33) were undetected by observer B, and 13.7% (n = 31) were undetected by observer A. The classification disagreement was 4.4% (n = 10) where an observer classified a target as a whale and the other observer classified it as uncertain. The whale agreement between both observers was 90.8% (n = 99).

There was 100% agreement between observers for all narwhals and belugas at the surface. All disagreements were individual targets that were perceived to be submerged resulting in darker coloration and where it was impossible to observe the whole outline body shape. The resolution of the images did not permit to differentiate nuances in color pattern between individuals or age classes. While juveniles are smaller than adults, we did not believe we could accurately distinguish adults from juveniles based on this sole criterion. Therefore, targets classified as whales could not be assigned as adults or juveniles.

In the 23 cropped images, the third satellite imagery observer detected 52 objects of interest with 45 categorized as belugas and seven as uncertain observations, whereas the experienced observers detected 50 objects of interest with 45 as a beluga whale and five as uncertain observations. The third observer detected two objects of interest that were not scored by the experienced observers and two observations were identified as uncertain by the third observer, but classified as whales by the experienced observers. Ultimately, the third observer had a 93% (n = 42) agreement with the experienced observers when detecting and categorizing an object as a beluga whale and 71% (n = 5) agreement categorizing an observation as uncertain.

**Fig 3:**
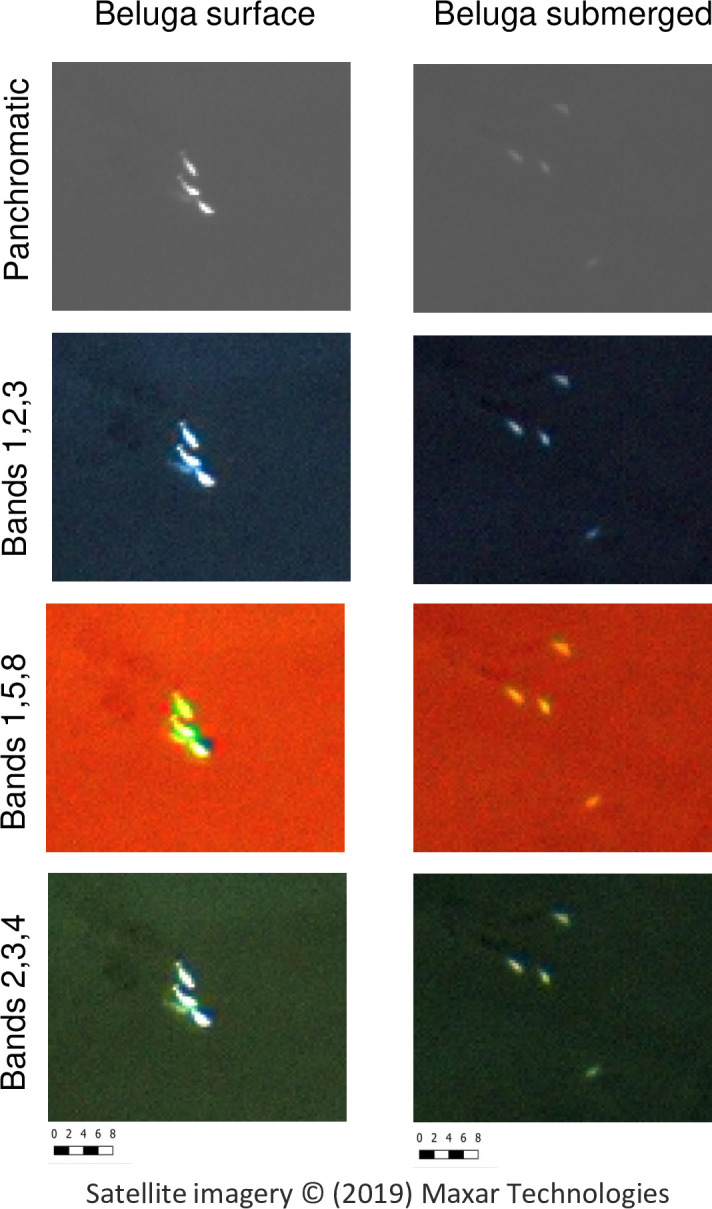
Panchromatic and pansharpened images of surface and submerged beluga whales from WorldView-3 imagery at 1:177 scale. Republished under a CC BY license, with permission from Maxar Technologies, original copyright 2019.

The BT algorithm performed the best of the three pansharenening methods tested on two subsections of the images; one subsection containing what observers perceived as mainly surface animals, and one subsection where animals were perceived to be submerged below the surface. Each subsection contained four individual belugas. Using the BT algorithm, all possibilities of three-band combinations were created. After visually inspecting all combinations, the three top performing combinations were selected. The band combinations a) 1, 2, 3 (coastal, blue, green); b) 1, 5, 8 (coastal, red and near-infrared 2); as well as c) 2, 3, and 4 (blue, green and yellow) (Fig 3) were most useful for detecting surface and submerged belugas. Our team preferred the combination a) for beluga detection, as these bands revealed the most contrast between the environment and the belugas making detection by observers faster and easier. However, all three band combinations could be helpful, especially for narwhals but we were unable to test pan sharpening algorithms on narwhals due to corruption of the multispectral data for these images.

## Discussion

This study contributes to our understanding of remote sensing and highlights the capabilities of using satellite imagery to detect marine mammals. While great whales have been detected using VHR satellite [2, 14], we provide here the first evidence that medium-size cetaceans can be detected using this method. Our results show that beluga and narwhal individuals can be reliably detected via VHR satellite by different observers. The variability in whale detection between observers remained consistent as environmental conditions varied (i.e. sea state (1–3), cloud cover (0–14%)). However, both expert observers noted that the detection rate of individuals in images with poor environmental conditions (i.e.; higher sea state, light see-through clouds) were lower than images with good visibility and low Beaufort sea states.

Identifying and counting beluga and narwhal from satellite imagery provides a promising mechanism for evaluating their distribution and abundance. The count estimates from this study are not representative of population estimates, as only a small portion of the Cumberland Sound beluga and Eclipse Sound narwhal summer ranges were covered. Moreover, to date, no availability bias correction factors exist for these species using VHR satellite imagery to account for whales that are unable to be detected since they are too deep in the water column. However, future studies could expand coverage and use satellite imagery for developing surface abundance estimates. Current abundance estimates for beluga and narwhal are developed from twin-otter aerial surveys [29, 37]. These surveys are expensive, require a large team of personnel, are challenging due to rapidly changing environmental conditions, and expose flight crew to potential aircraft failure and crash. Finally, aerial surveys may disturb animals if flown at low elevations [40]. Satellite imagery, however, also has limitations as feasibility of capturing clear images can be low as a result of weather or position on the planet. Obtaining full coverage of an entire populations range within a few days can be cost-prohibitive and logistically difficult depending on the orientation of the satellites orbit in relation the species range (i.e. a satellite with a north-south orbit has a better chance of photographing a north-south fiord (i.e. Tremblay Sound in our case) all in one day, compared to an east-west fiord such as Clearwater Fiord, which took days to photograph), and the cloud cover. If an area cannot be photographed within a single day, animals can move [35], and therefore you may duplicate or miss them. However, this is also a limitation with traditional aerial surveys which can take weeks due to inclement weather (i.e. high winds or low cloud cover) [37]. Before satellite imagery can be used to estimate abundance for the population two sources of information are needed; satellite imagery of the entire summer distribution, and information on the depth at which animals can be seen from the satellite imagery under various environmental conditions (i.e. varying sea states and water turbidity [41, 42]) in order to adjust surface estimates to account for animals that are diving (referred to as availability bias). An approach to determine availability bias would be to develop spectral reflectance profile above the surface for each whale species and then placing whale dummies made of panels with the same spectral reflectance of the targeted species above the surface in different conditions and depths to develop adjustment factors for satellite imagery [43–46]. In addition, having a spectral library of different atmospheric and water turbidity conditions would help understanding the exact environment conditions at the time and location of image acquisition to adapt the correction factors [47].

In this study we are confident that only one species was present in each study area as assessed by field programs in Tremblay Sound in 2017 and Cumberland Sound in 2019. Thus, we were able to avoid misclassification of species. However, the morphological similarity between the species make them indistinguishable to human observers using panchromatic satellite images. Use of pansharpened algorithms and tailored bands may provide more detail for differentiating the species from one another in the future, which could allow this method to be used in areas where the two species might overlap [48]. As satellite technology improves (e.g. finer resolution) it may increase our likelihood to differentiate species. Finally, VHR imagery only provides information form a snap-shot in time, and does not provide longer term data on distribution that can be gathered from telemetry or acoustic data.

Despite success using satellite imagery to count individuals of different species, this method is still in its infancy in terms of using it as a monitoring tool. As any other monitoring or survey method, satellite imagery comes with its own limitations and challenges, primarily, clear environmental conditions are required [49]. For instance, we found that to detect medium sized cetaceans from space, environmental conditions needed to be optimal with calm waters with no cloud cover (even thin transparent cloud cover reduced detectability) [2, 14]. Targets need to be of sufficient size to be detected at the resolution provided by the satellite [49]. Currently the best commercially available VHR resolution is 0.31 m, but as technology improves smaller targets will be detectable, which will increase the versatility of this observation method. There should also be a colour contrast between the landscape and detected target [49], which means this method is less effective for cryptic species. In areas where multiple species exist, ground-truthing observations is needed to support species identification from satellites [49]. Furthermore, for monitoring in the marine environment, VHR imagery is not routinely acquired for the open ocean which means to gather information for these areas the satellite must be tasked which comes at a much greater expense. Thus, depending on budgetary limitations, the feasibility of using this technology may be dependent on the species and their movement outside of more coastal areas. Finally, VHR imagery only provides information from a snap-shot in time, and does not provide longer term data on distribution that can be gathered from telemetry or acoustic data.

One of the many challenges of using satellite imagery for cetacean monitoring is the extensive time requirement for readers to process the images. Cubaynes et al. [2] reported an approximate time of 2 mins per km^2^ at a scale of 1:1,500 whereas in our study it took ~2.5 mins to scan 1 km^2^ at a scale of 1:536. Therefore, the next step in the use of VHR imagery for cetacean monitoring is automated detection of whales from space. The spectral difference between whales and the environment from radiometrically corrected imagery provides an opportunity to automate whale identification. Hurdles in automation include; false positives where other features resemble whales such as ice, waves, or rocks; whales that are slightly below the surface changing the apparent colour of the whale compared to surface individuals; and varying environmental conditions Additional information, such as the size and shape of the object, as well as texture differences from the surrounding environment can be used to increase detection rates, while minimizing false positives [49]. A number of these techniques are currently being explored to investigate the development of algorithms for a range of different species [50, 51].

As the Arctic continues to experience unprecedented climatic changes, the probability of obtaining clear VHR imagery may be lower [52]. However, these changes will ultimately make any visual method to detect and survey marine mammals in the Arctic challenging. The emergence of new platforms such as VHR satellite imagery could become even more important for taking advantage of short windows of opportunity to monitor Arctic cetaceans populations over vast areas, while also reducing risks to survey observers.

## Conclusion

In recent years, space-based solutions have become more popular for monitoring wildlife populations [49]. This new method has the potential to offer a faster, safer, non-invasive and environmentally friendly alternative platform to study marine mammals than traditional aerial surveys [2, 53]. Once validated for specific species, the use of VHR satellite imagery could be incorporated into adaptive management and monitoring plans, especially in remote areas such as the Arctic. In the future, the launch of new satellites to replace older ones will make Very high-resolution imagery more accessible and the cost per scene will be reduced. However, the actual spatial resolution available will not become much more finer since these satellites are already at the sub-metre resolution [46]. Improvements in analytical techniques are occurring, such as deep machine learning which will allow analysts to derive more information from the very high-resolution imagery.

## Supporting information

S1 Table WorldView-3 satellite images (36cm resolution) taken in August 2019 in Clearwater Fiord, Nunavut and August 2017 in Tremblay Sound, Nunavut. All images were acquired from L3Harris Geospatial.
(TIF)

S1 Database (CSV)

S2 Database (CSV)
