# Correction: Mapping Arctic cetaceans from space: A case study for beluga and narwhal

**DOI:** 10.1371/journal.pone.0258238

**Published:** 2021-09-30

**Authors:** The PLOS ONE Staff

There is information missing from the Competing Interests statement. The correct, complete Competing Interests statement is as follows: The authors have read the journal’s policy and have the following competing interests: BC and ET are employees of Whale Seeker. These authors own the company and led the research, analysis, and writing of this article. This does not alter our adherence to PLOS ONE policies on sharing data and materials. There are no patents, products in development or marketed products associated with this research to declare.

The publisher apologizes for the error.
