# Supplementary figures and images for: Mapping Arctic cetaceans from space: A case study for beluga and narwhal
Source: PLoS One. 2021 Aug 4;16(8):e0254380. doi: 10.1371/journal.pone.0254380 (PMC8336832; doi:10.1371/journal.pone.0254380)

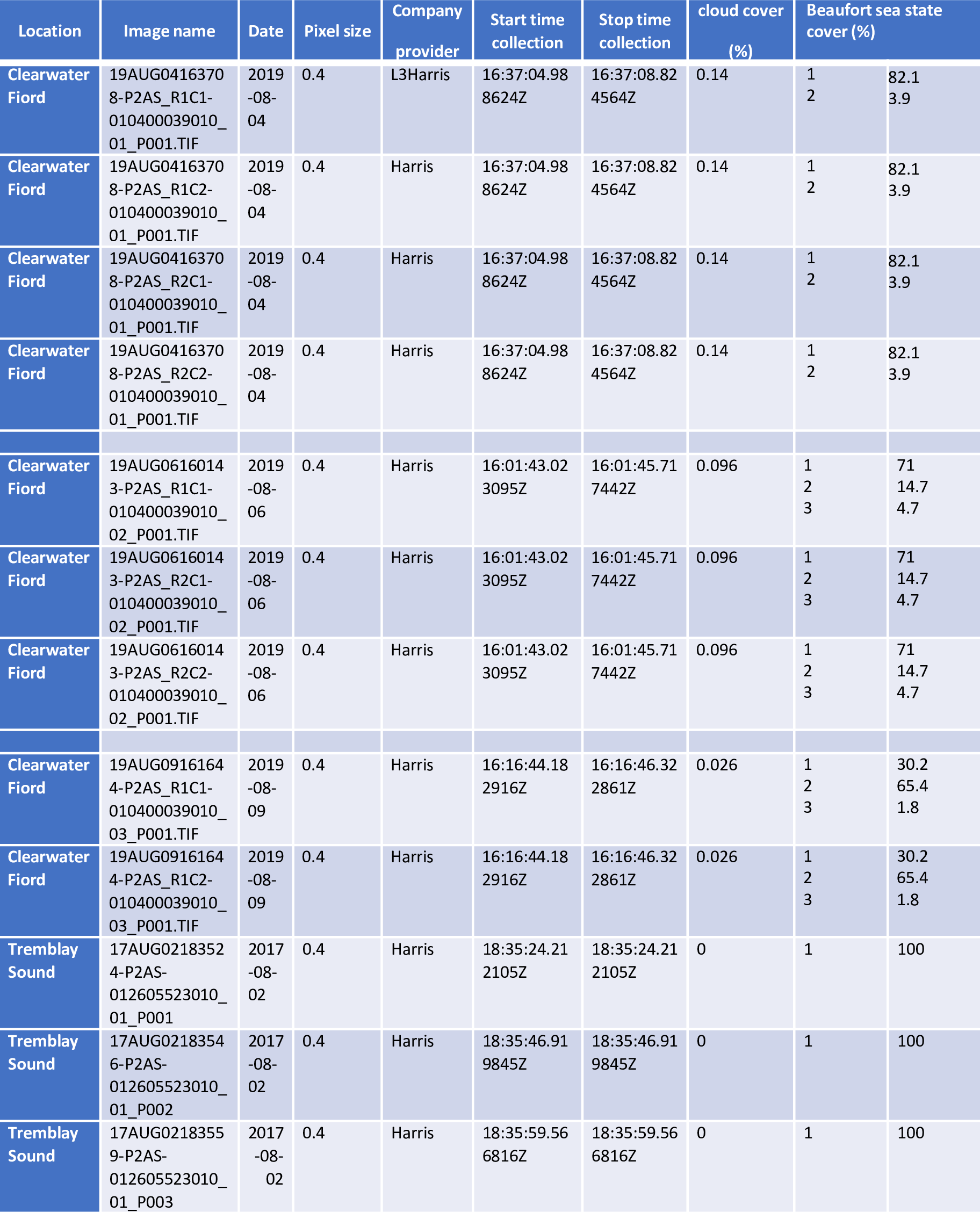

Supplement: S1 Table — All images were acquired from L3Harris Geospatial. (TIF) [file pone.0254380.s001.tif]
